# Clinical Laboratory Results as Prognosis Marker in Advanced Stage Non-small Cell Lung Cancer (NSCLC) in Indonesia

**DOI:** 10.7759/cureus.29386

**Published:** 2022-09-20

**Authors:** Arif R Hanafi, Achmad M Jayusman, Noorwati Sutandyo, Sri Kurniawati, Lyana Setiawan, Alyssa Diandra, Kusmantoro Hidayat

**Affiliations:** 1 Pulmonology, Dharmais Cancer Hospital, Jakarta, IDN; 2 Division of Hematology and Medical Oncology, Dharmais National Cancer Hospital, Faculty of Medicine, University of Indonesia, Jakarta, IDN; 3 Internal Medicine, Dharmais Cancer Hospital, Jakarta, IDN; 4 Clinical Pathology, Dharmais Cancer Hospital, Jakarta, IDN; 5 General Medicine, Dharmais Cancer Hospital, Jakarta, IDN

**Keywords:** nsclc, internal medicine, pulmonology, survival, clinical laboratory

## Abstract

Background: Lung cancer is the most common cause of cancer-related death among men in the world. Given the very high mortality caused by lung cancer, a biological marker to determine a more sensitive therapy among non-small cell lung cancer (NSCLC) patients is needed. This study aims to demonstrate that the clinical laboratory result can be a prognosis marker in NSCLC patients in Indonesia.

Methods: This study was a retrospective cohort study. The sample was obtained from the patient's serum and the examined routine blood test (hemoglobin, leukocyte, platelets), hemostasis (fibrinogen and D-dimers), blood chemistry test (aspartate transaminase [AST], alanine transaminase [ALT], albumin, urea, creatinine, and blood glucose), electrolyte (sodium, potassium, chloride, and calcium) and tumor markers (carcinoembryonic antigen [CEA] and Cyfra 21-1). Data were analyzed and interpreted using SPSS (IBM Corp., Armonk, NY, USA). Analysis of the data was done to find the survival rate of sociodemographic variables, clinicopathologic variables, and clinic laboratory variables.

Results: The study findings showed statistically significant results that were poor prognosis for these following conditions: performance status (PS) 3-4 median survival (MS):26 days, p=<0.001; TNM stage IVb MS:58 days, p=0.001; high leukocyte MS:69 days, p=0.018; low platelet MS:50 days, p=0.013; high D-dimer MS:69 days, p=0.020; low albumin MS:56 days, p=0.001; high sodium MS:15 days, low sodium MS:50 days, p=<0.001; high chloride MS:15 days, low chloride MS:27 days, p=<0.001.

Conclusion: In the advanced stage NSCLC, these findings indicate poorer prognoses; PS 3-4, IVb clinical stage, leukocytosis, thrombocytopenia, hyper-coagulopathy, hypoalbuminemia, hyper-hyponatremia, and hyper-hypochloremia. Further studies regarding the correlation between clinical laboratory and survival rate are needed.

## Introduction

Lung cancer is the most common cause of cancer-related deaths among men in the world [1,2]. According to data from Dharmais National Cancer Hospital, lung cancer is the leading cause of cancer-related death among men (28.94%). The high mortality of lung cancer is due to the disease’s advanced stage and the treatment resistance [3].

Given the extremely high death rate from lung cancer, it is necessary to identify a molecular marker in non-small cell lung cancer (NSCLC) patients in order to establish a more sensitive course of treatment [4,5]. Some biological markers have become predictive and prognostic markers for the NSCLC. Predictive biological markers indicate therapeutic effectiveness because there is an interaction between the marker and the therapy on the therapeutic outcome. Prognostic biology indicates the survival of the patient regardless of the therapy given because biology is an indicator of the aggressiveness of the tumor itself.

Biological markers now are quite diverse; from ALK fusion oncogenes, reactive oxygen species 1 (ROS1) gene rearrangements, sensitizing epidermal growth factor receptor (EGFR) mutations, and programmed cell death ligand 1 (PD-L1). The presence of exon 19 deletions in the EGFR gene or mutations in exon 21 L858R are considered predictive for management with EGFR tyrosine kinase inhibitors (EGFR-TKI), for example with gefitinib, erlotinib, and afatinib [6,7]. These mutations are often called sensitizing EGFR mutations. However, it has not been demonstrated that the presence of exon 19 deletions of the leucin-arginin-glutamate-alanin (LREA) type or exon 21 L858R mutations is a predictive marker of the survival of patients with NSCLC in the absence of differentiated therapy [8].

The estimated prevalence of NSCLC patients with EGFR mutation in Asia was 41.9%. The most frequent mutations were exon 19 deletions (49.2%) and L858R mutations in exon 21 (41.1%) [9]. EGFR gene mutations are associated with several characteristics such as mutations more often in female patients, adenocarcinoma type NSCLC, non-smoker/smoking cessation patients, and East Asian patients [10,11]. Overexpression of EGFR is associated with a poor prognosis in the NSCLC [12,13]. EGFR mutations occur in about 10% of Caucasian race patients while Asian patients are 62% [14].

Laboratory results can also indicate a poor prognosis in NSCLC. Several studies discovered laboratory findings such as a high prevalence of anemia in lung cancer correlates with its clinical stage, which affects quality of life prognosis and therapeutic outcomes [15]. Leukocytosis and anemia have been associated with survival as a prediction of a poor prognosis for NSCLC [16]. Hyper-coagulopathic status in malignancy occurs because of the ability of cancer cells to activate the coagulation system, so hypercoagulation is very high in cancer and affects mortality and morbidity [17]. The increase in carcinoembryonic antigen (CEA) of 37.1% and cytokeratin-19 fragments 21-1 (Cyfra 21.1) of 68.5% has sensitivity and diagnostic value in the NSCLC [18]. Increased CEA and Cyfra 21.1 become negative predictors of the NSCLC [19].

Leukocytosis found in lung cancer can occur due to infection, steroid use or hematopoietic growth factor, bone marrow problems, or paraneoplastic manifestations. This may indicate poor prognosis and aggressiveness of the disease [20]. Tumor-related leukocytosis and cytokine production often occur in the course of lung cancer, a phenomenon that causes worsening patient prognosis [21]. Leukocytosis and anemia have been associated with poor prognosis for NSCLC [22].

Hypoalbuminemia can be an independent negative prognostic factor for the occurrence of recurrence and poorer survival in the post-operative stage 1 NSCLC [23]. Initial assessment of albumin levels and systemic inflammatory response (SIR) of NSCLC patients have a prognostic role. Improving albumin levels has a beneficial effect before the patient is given chemotherapy [24]. Hypercoagulopathy status in malignancy occurs because of the ability of cancer cells to activate the coagulation system, so hypercoagulation often happens in cancer and affects mortality and morbidity [25].

Hyponatremia in oncology is a negative prognosis factor because this occurs due to syndrome of inappropriate antidiuretic hormone (SIADH), causing excessive production of arginine vasopressin by tumors or anticancer effects. Hyponatremia can also occur due to diarrhea and vomiting due to cancer therapy, hyponatremia is always followed by hypochloremia. The importance of electrolyte laboratory tests before therapy is given [26]. In India, NSCLC patients have a high risk of hyponatremia and this is associated with a poor prognosis [27]. Hyponatremia is not only a sign of the prognosis of NSCLC therapy but also predictive of the efficacy of erlotinib therapy, as a simpler and cheaper biomarker [28]. Cancer patients often experience abnormalities in their serum electrolytes: hyponatremia, hypokalemia, hyperkalemia, hypophosphatemia, and hypercalcemia. These electrolyte disturbances are not exclusively caused by underlying cancer but is a sign of a paraneoplastic process that leads to poor prognosis and poor quality of life. Early recognition of the disorder and correcting this electrolyte disturbance are very important in cancer management [29].

This study aims to prove that laboratory test results can be a predictive biological marker for prognosis in NSCLC patients in Indonesia. This study also aims to prove that laboratory test results are related to clinical status as well as histopathology in NSCLC patients and to find the correlation between laboratory test value with survival rate, performance status, metastasis M1b, and cancer cell type. This study was previously presented as a meeting abstract at The 25th Congress of the Asian Pacific Society of Respirology 2021 on 20 November 2021.

## Materials and methods

Study design

This study was a retrospective cohort study. The data was collected from medical record of NSCLC patients in Dharmais National Cancer Center, Indonesia. 

Sample number and criteria

The research subjects were 52 patients who came for treatment at Dharmais Hospital National Cancer Center in 2017 and met the inclusion criteria. Inclusion criteria were NSCLC patients who were proven by the anatomic pathology examination results from both the Dharmais Hospital National Cancer Center and other hospitals, were equal to or above 18 years and had not received treatment. The exclusion criteria were subjects who had a primary tumor in another organ and refused to participate in research.

Sample examination

The sample was obtained from the patient's serum. As much as 5 ccs of the patient's blood was drawn and then centrifuged at a speed of 1800 g for 10 minutes. The serum obtained was separated into cryotubes and stored in a cold cabinet at -80°C. Samples were taken concurrently when the patients were undergoing a lung cancer diagnosis. Consequently, the routine blood test (hemoglobin, leukocyte, platelets), hemostasis (fibrinogen and D-dimer), blood chemistry (aspartate transaminase [AST], alanine transaminase [ALT], albumin, urea, creatinine, and blood glucose), electrolytes (sodium, potassium, chloride, and calcium) and tumor markers (carcinoembryonic antigen [CEA] and Cyfra 21-1) were examined.

Tools and materials

The tools used were 1.5ml cryotube; sterile Eppendorf tubes (1.5ml; 500ml; 200ml); micro pipettes 0.1-2.5ml, 0.5-10ml, 2ml; 100-1000ml (Eppendorf, Hamburg, Germany); centrifugation, glassware (Duran, Wertheim/Main, Germany, and Pyrex, Stoke-on-Trent, UK); filtered tips 10ml; filtered tips 100ml, filtered tips 1000ml, yellow tips 200ml, vortex.

Study procedures

Data collection was carried out after the researchers received approval (no 032/KEPK/V/2016) from the Ethics Committee of Dharmais National Cancer Center, Indonesia. The research subjects were taken consecutively following the research criteria to meet the number of research samples. Subjects who agreed to participate in the research then signed the informed consent. The sample was 5 cc of blood taken from the patients. Samples were taken concurrently when the patient was undergoing lung cancer diagnosis.

Subjects were followed until completing target therapy treatment for three months and The Response Evaluation Criteria in Solid Tumors (RECIST) was then evaluated. Patients who belonged to the anti-EGFR resistant were patients who experience progression (RECIST status is PD [Progressive Disease]). Patient demographic data were collected from the patient's medical record.

The research subjects were stage IIIb and IV of NSCLC who were proven through anatomic pathology examination (the patients were examined in the anatomical pathology department to determine the stage of NSCLC at the Dharmais Cancer Hospital or at another hospital. They were categorized into two groups: squamous cell carcinoma and non-squamous cell carcinoma (adenocarcinoma and large cell).

After signing the informed consent, about 5 ccs of the patient's blood was drawn and taken to the Clinical Pathology laboratory of the Dharmais Cancer Hospital and the routine blood (hemoglobin, leukocyte, platelets), hemostasis (fibrinogen and D-dimers), blood chemistry (AST and ALT, albumin, urea, creatinine, and blood glucose), electrolyte (sodium, potassium, chloride, and calcium) and tumor markers (CEA and Cyfra 21-1) tests were then run. Blood was taken for laboratory examination. Patients with NSCLC in their anatomic pathology results had their EGFR gene mutations tested. The evaluation in this study was a clinical picture of overall survival from routine blood, hemostasis, blood chemistry, electrolyte, and tumor markers.

Statistical analysis of study

Data were analyzed using Statistical Package for Social Sciences (SPSS; IBM Corp., Armonk, NY, USA) and interpreted using descriptive analysis (presented by tables and graphs), and bivariate analysis (using Mann Whitney-U for those two types of variables or Kruskal-Wallis for those more than two types of variables). Kaplan-Meier analysis was used to find out the association between patients' characteristics which were sociodemographic, clinicopathologic, performance status, and their correlation with survival rate. This study used the significance p-value of <0.05 and a confidence interval of 95%.

## Results

A total of 52 patients were included in the study. There were 71.2% men with mean age of all patients was 55.87 ± 11.47 years old. The youngest patient was 39 and the oldest one was 72. There were 7.7% patients included in the age group of <40 years old, 53.8% patients included in 40-60 years old, and 38.5% patients included in >60 years old. Of those patients, as many as 46.2% patients were active smokers. Performance status score was divided into four categories, which were performance status (PS) 3-4 comprising 26.9% of patients, PS 2 comprising 36.5% of patients, PS 1 comprising 9.6% of patients, and PS 0 comprising 26.9% of patients. Most of the patients were in stage IV (94.3%) of which 55.8% were in stage IV B and had adenocarcinoma (82.7%) as the cancer cell type. 80.8% of patients had negative EGFR mutations (Table 1).

**Table 1:** Sociodemographic and clinicopathology variables in advanced-stage non-small cell lung cancer (NSCLC) patients ECOG, Eastern Cooperative Oncology Group; TNM, Tumor, Node, and Metastasis; EGFR, epidermal growth factor receptor

Variable	n	%
Sex		
Male	37	71.2
Female	15	28.8
Age (years), mean (SD)	55.87 (±11.47)	
Age Group		
<40 years old	4	7.7
40-60 years old	28	53.8
>60 years old	20	38.5
Smoking		
Yes	24	46.2
No	28	53.8
ECOG Performance Status (PS)		
PS 3-4	14	26.9
PS 2	19	36.5
PS 1	5	9.6
PS 0	14	26.9
TNM Stage (n = 52)		
IVb	29	55.8
IVa	20	38.5
IIIb	3	5.7
Type of cancer cell		
Adenocarcinoma	43	82.7
Squamous cell carcinoma	9	17.3
EGFR mutation status		
Positive	10	19.2
Exon 18	1	1.9
Exon 19	5	9.6
Exon 20	0	0
Exon 21	2	3.8
Double mutation	2	3.8
Negative	42	80.8

From the study it was obtained that 65.4% of patients had anemia, 86.5% had leukocytosis, 61.5% had high fibrinogen level, 86.5% had high D-dimer level, and 82.7% had high CYFRA 21-1 level (Table 2).

**Table 2:** Clinical laboratory variables in advanced-stage non-small cell lung cancer (NSCLC) patients AST, aspartate aminotransferase; ALT, alanine transaminase; CEA, carcinoembryonic antigen; Cyfra 21-1, Cytokeratin-19 Fragments 21-1

Variables	n	%
Hemoglobin (g/dL)		
>16	0	0
M: 13-16, F: 12-16 (Normal)	18	34.6
M: <13, F:<12	34	65.4
Leukocyte (per mm^3^)		
> 10,000	45	86.5
3,200-10,000 (Normal)	7	13.5
< 3,200	0	0
Platelets (per mm^3^)		
> 380,000	20	38.5
170,000-380,000 (Normal)	29	55.8
< 170,000	3	5.8
Fibrinogen (mg/dL)		
> 400	32	61.5
200-400 (Normal)	20	38.5
D-Dimer (ng/dL)		
≥300	45	86.5
<300 (Normal)	7	13.5
AST (µ/L)		
>35	7	13.5
5-35 (Normal)	45	86.5
ALT (µ/L)		
> 35	8	15.4
5-35 (Normal)	44	84.6
Albumin (mg/dL)		
3.5-4.5 (Normal)	32	61.5
< 3.5	20	38.5
Urea (mg/dL)		
> 20	9	17.3
9-20 (Normal)	43	82.7
Creatinine (mg/dL)		
> 1.3	6	11.5
0.6-1.3 (Normal)	46	88.5
Blood glucose (mg/dL)		
≥140	5	9.6
<140 (Normal)	47	90.4
Sodium (mmol/L)		
> 144	1	1.9
135-144 (Normal)	33	63.5
< 135	18	34.6
Potassium (mmol/L)		
> 4.8	1	1.9
3.6-4.8 (Normal)	46	88.5
< 3.6	5	9.6
Chloride (mmol/L)		
> 106	1	1.9
97-106 (Normal)	42	80.8
< 97	9	17.3
Calcium (mmol/L)		
> 10.4	3	5.8
8.8-10.4 (Normal)	42	80.8
< 8.8	7	13.5
Tumor marker		
CEA (ng/mL)		
≥3	29	55.8
<3 (Normal)	23	44.2
Cyfra 21-1 (ng/mL)		
≥3.3	43	82.7
<3.3 (Normal)	9	17.3

Twenty-eight patients died during one year of follow-up. Based on Kaplan-Meier table, PS and TNM stage were significantly associated with survival rate. PS 3-4 (HR 3.65, p<0.001) and TNM stage IV B (HR 2.993, 95% CI: 1.634-5.265, p=0.001) were significantly associated with poor prognosis in advanced stage NSCLC patients. Median survival for PS 3-4 and TNM stage IV B was 26 days and 28 days, respectively (Table 3).

**Table 3:** Analysis of Kaplan-Meier's survival for sociodemographic and clinicopathology variables in advanced-stage non-small cell lung cancer (NSCLC) patients ECOG, Eastern Cooperative Oncology Group; TNM, Tumor, Node, and Metastasis; EGFR, epidermal growth factor receptor

Variables	n	Median Survival (day)	Survival Rate (1 year)		HR	95% CI	p
			n	%			
Sex							
Male	37	81	16	43.2	0.832	(0.393-1.760)	0.831
Female	15	244	8	53.3			
Age group							
<40 years	20	55	2	50.0	1.007	(0.978-1.038)	0.944
40-60 years	28	176	11	39.3			
>60 years	4	74	11	55.0			
Smoking							
Yes	3	70	8	33.3	1.732	(0.906-3.311)	0.132
No	49	244	16	57.1			
ECOG Performance Status (PS)							
PS 3-4	14	26	6	42.9	3.65	-	<0.001
PS 2	19	68	4	21.1	2.546	(1.186-5.463)	
PS 1	5	176	3	60.0	0.755	(0.263-2.166)	
PS 0	14	>365	11	78.6	0.073	(0.023- 0.231)	
TNM Stage							
IVb	29	58	8	27.6	2.993	(1.634-5.265)	0.001
IVa	20	263	13	65.0			
IIIb	3	>365	3	100.0			
Type of cancer cell							
Adenocarcinoma	43	81	22	51.2	0.857	(0.378-1.940)	0.563
Squamous cell carcinoma	9	92	2	22.2			
EGFR gene mutation status							
Positive	10	81	5	50	0.421	(0.160-1.102)	0.342
Negative	42	70	19	45.2			

Kaplan-Meier table for clinical laboratory values showed high leukocyte, low platelet, high D-dimer, low albumin, sodium and chloride levels were associated with poor prognosis of survival rate in advanced stage NSCLC patients (Table 4).

**Table 4:** Analysis of Kaplan-Meier's survival for laboratory variables in advanced-stage non-small cell lung cancer (NSCLC) patients AST, aspartate aminotransferase; ALT, alanine transaminase; CEA, carcinoembryonic antigen; Cyfra 21-1, Cytokeratin-19 Fragments 21-1

Variables	n	Median Survival (day)	Survival Rate (1 year)		HR	95% CI	p
			n	%			
Hemoglobin (g/dL)							
M:13-16, F:12-16 (Normal)	18	244	11	61.1	0.993	(0.981-1.005)	0.095
M: <13, F: <12	34	68	13	38.2			
Leukocyte (per mm^3^)							
> 10,000	45	69	18	40.0	1.000	(1.000-1.001)	0.018
3,200-10,000 (Normal)	7	>365	6	85.7			
Platelets (per mm^3^)							
> 380,000	20	263	12	60.0	0.999	(0.997-1.001)	0.013
170,000-380,000 (Normal)	29	70	12	41.4			
< 170,000	3	50	0	0.0			
Fibrinogen (mg/dL)							
> 400	32	263	17	53.1	1.000	(0.998-1.000)	0.189
200-400	20	69	7	35.0			
D-dimer (ng/mL)							
≥300	45	69	18	40.0	1.000	(0.999-1.000)	0.020
<300 (Normal)	7	>365	6	85.7			
AST (µ/L)							
> 35	7	81	1	14.3	1.000	(1.000-1.010)	0.432
5-35 (Normal)	45	92	23	51.1			
ALT (µ/L)							
> 35	8	56	1	12.5	1.007	(0.997-1.017)	0.147
5-35 (Normal)	44	92	23	52.3			
Albumin (mg/dL)							
3.5-4.5 (Normal)	32	263	20	62.5	0.939	(0.878-1.000)	0.001
< 3.5	20	56	4	20.0			
Urea (mg/dL)							
> 20	9	68	4	44.4	1.009	(0.994-1.026)	0.815
9-20 (Normal)	43	81	20	46.5			
Creatinine (mg/dL)							
> 1.3	6	>365	5	83.3	0.991	(0.980-1.001)	0.070
0.6-1.3 (Normal)	46	74	19	41.3			
Blood glucose (mg/dL)							
≥140	5	39	1	20.0	1.002	(0.996-1.000)	0.068
<140 (Normal)	47	92	23	48.9			
Sodium (mmol/L)							
>144	1	15	0	0	0.965	(0.901-1.034)	<0.001
135-144 (Normal)	33	263	19	57.6			
< 135	18	50	5	27.8			
Potassium (mmol/L)							
> 4.8	1	56	0	0	0.973	(0.919-1.030)	0.394
3.6-4.8 (Normal)	46	92	22	47.8			
< 3.6	5	50	2	40.0			
Chloride (mmol/L)							
> 106	1	15	0	0.0	0.993	(0.969-1.018)	<0.001
97-106 (Normal)	42	244	21	50.0			
< 97	9	27	3	33.3			
Calcium (mmol/L)							
> 10.4	3	92	2	66.7	1.005	(0.983-1.029)	0.508
8.8-10.4 (Normal)	42	176	20	47.6			
<8.8	7	56	2	28.6			
CEA (ng/mL)							
≥3	29	70	14	48.3	0.999	(0.999-1.000)	0.652
<3 (Normal)	23	92	10	43.5			
CYFRA 21-1 (ng/mL)							
≥3.3	43	70	19	44.2	1.000	(0.999-1.000)	0.233
<3.3 (Normal)	9	299	5	55.6			

## Discussion

Lung cancer is the most common cancer. Based on population-based cancer registration in Jakarta (2005-2007), lung cancer ranks first of all cancer cases that occur in men (ASR = 11.7). In women, lung cancer is the fourth highest cancer (ASR = 4.5) [3]. According to Hospital-Based Cancer Registration (2003-2007) data in Dharmais, lung cancer in men ranked second (13.4%) but lung cancer deaths took the top spot (18.48%). In women, lung cancer ranked seventh (2.82%) while lung cancer deaths were the fifth (5.52%) [30].

Smoking as the main risk factors contributes to the highest mortality rate in cancer [31,32]. Cigarette smoke contains a lot of carcinogenic (e.g., nitrosamines, benzopyrene diol epoxide) [33]. The risk of lung cancer increases with the increasing number of cigarettes smoked per day. Passive smokers also have a higher relative risk (RR = 1.24) for lung cancer from secondhand smoke [34-36].

The high prevalence of anemia in lung cancer correlates with its clinical stage and this affects the quality of life of the prognosis and therapeutic outcome [15]. Leukocytosis and anemia have been associated with survival as a prediction of a poor prognosis for NSCLC [22]. Hyper-coagulopathic status in malignancy occurs because of the ability of cancer cells to activate the coagulation system, so hypercoagulation is very high in cancer and affects mortality and morbidity [25]. The increase in CEA of 37.1% and Cyfra 21.1 of 68.5% had sensitivity and diagnostic value in the NSCLC [18]. Increased CEA and Cyfra 21.1 became a negative predictor of the NSCLC [19].

In this study, the most advanced NSCLC stage obtained was T4 N2 M1b stage IVb multiorgan metastasis with positive EGFR gene mutation (19.2%) and exon 19 (9.6%). The increasing clinical stage of the prognosis is poor, this classification of stages is very useful for determining prognosis and therapeutic strategies [37,38]. The estimated prevalence of NSCLC patients with EGFR mutation in Asia was 41.9%. The most frequent mutations were exon 19 deletions (49.2%) and L858R mutations in exon 21 (41.1%) [9]. EGFR gene mutations were associated with several characteristics such as mutations more frequently in female patients, adenocarcinoma type NSCLC, non-smokers / those who had quitted smoking, and East Asian patients [10,11]. Overexpression of EGFR is associated with a poor prognosis in the NSCLC [12,13].

The results of this study the advanced stage NSCLC survival analysis obtained statistically significant results as a poorer prognosis for several conditions, namely ECOG Performance Status (PS) 3-4, leukocytosis, thrombocytopenia, hyper-coagulopathy, hypoalbuminemia, hyponatremia, hypochloremia, IVb clinical stage, and M1b metastasis.

Leukocytosis often found in lung cancer can occur due to infection, steroid use or hematopoietic growth factor, bone marrow problems, or paraneoplastic manifestations, this is related to poor prognosis and aggressiveness of the disease [20]. Tumor-related leukocytosis and cytokine production often occur in the course of lung cancer, a phenomenon that causes worsening patient prognosis [21]. Leukocytosis and anemia have been associated with survival as a prediction of a poor prognosis for NSCLC [22].

Hypoalbuminemia can be an independent negative prognostic factor for the cancer recurrence and poorer survival in the post-operative stage 1 NSCLC [23]. Initial assessment of albumin levels and Systemic Inflammatory Response (SIR) of NSCLC patients has a prognostic role. Improving albumin levels has a beneficial effect before the patient is given chemotherapy [24].

Hyper-coagulopathic status in malignancy occurs because of the ability of cancer cells to activate the coagulation system, so hypercoagulation often happens in cancer and affects mortality and morbidity [25].

Hyponatremia in oncology is a negative prognosis factor because this occurs due to Syndrome of Inappropriate Antidiuretic Hormone (SIADH) excessive production of arginine vasopressin by tumors or anticancer effects. Hyponatremia can also occur due to diarrhea and vomiting as a side effect of the cancer therapy. Hyponatremia is always followed by hypochloremia. Therefore, it is important to run electrolyte laboratory tests before the therapy is given [26]. In India, NSCLC patients have a high risk of hyponatremia and this is associated with a poor prognosis [27]. Hyponatremia is not only a sign of the prognosis of NSCLC therapy but also predictive of the efficacy of erlotinib therapy, as a simpler and cheaper biomarker [28].

Cancer patients often experience abnormalities in their serum electrolytes: hyponatremia, hypokalemia, hyperkalemia, hypophosphatemia, and hypercalcemia. This electrolyte disturbance is not exclusively caused by underlying cancer but is a sign of a paraneoplastic process that causes prognosis and poor quality of life. Early recognition of the disorder and correcting this electrolyte disturbance are very important in cancer management [29].

The cohort retrospective design of this study showed more strength in clinical laboratory value as survival risk factor. However, regimen of therapy, comorbidities, and other treatment modalities were not available in this study while these might influence the clinical laboratory value and survival rate.

## Conclusions

In this study, laboratory test results are proved to have correlation with survival rate in advanced-stage NSCLC. Several laboratory findings indicate poor prognoses, especially PS 3-4, IVb clinical stage, leukocytosis, thrombocytopenia, hyper-coagulopathy, hypoalbuminemia, hyper-hyponatremia, and hyper-hypochloremia. This study has shown the importance of laboratory results in obtaining better survival rates. Further studies regarding the correlation between clinical laboratory and survival rate are still needed.
